# The impact of protective measures against COVID-19 on the wellbeing of residents in nursing homes and their relatives: a rapid review

**DOI:** 10.1186/s12877-023-04300-7

**Published:** 2023-10-11

**Authors:** P. Schneider, M. Abt, C. Cohen, N. Marmier, C. Ortoleva Bucher

**Affiliations:** 1 La Source School of Nursing, HES-SO University of Applied Sciences and Arts Western Switzerland, Lausanne, Switzerland; 2 Faculty of Biology and Medicine, Institute of Higher Education and Research in Health Care (IUFRS), Lausanne, Switzerland

**Keywords:** Covid-19, Protective measures, Nursing homes, Residents, Relatives, Social isolation, Wellbeing, Quality of life

## Abstract

During the COVID-19 pandemic and compared to other population groups, older people were at a heightened risk of developing lethal disease symptoms. Thus, many countries put in place protective measures to protect this “at-risk” population, especially in nursing homes, to limit the number of infections. These measures consisted mostly in the absence of social gatherings, the impossibility for relatives to enter the nursing homes, limitations in group activities, and the ban of group-eating. Although these measures were helpful to mitigate the spread of the disease, they also induced long periods of social isolation for the residents of nursing homes. This could have impacted the wellbeing and quality of life of residents and their relatives, with a possible impact on the overall health of residents. We designed this rapid review to investigate the literature on the impact of COVID-19 protective measures in nursing homes on the quality of life, wellbeing and physical health of residents and their relatives. Our results showed that most of the articles included in the review, either using qualitative or quantitative methods, evidenced a detrimental impact of protective measures on resident’s and their relatives’ wellbeing. We argue that, in the event of a new pandemic similar to the COVID-19, protective measures should also take into account their psychological impact, and not only their physical impact.

**Supplementary Information:**

The online version contains supplementary material available at 10.1186/s12877-023-04300-7.

## Introduction

First identified in December 2019, the COVID-19 disease outbreak was declared a pandemic by the World Health Organization in March 2020. In face of the health risk imposed by this disease, many countries put protective measures in place for at-risk populations. Older people were especially at risk of developing fatal symptoms, compared to other population groups [7]. Thus, measures were taken in nursing homes to minimize the number of people contracting the disease. These measures aimed primarily to minimize contact with persons outside the nursing homes and to restrict movement (room isolation, stopping of group activities, group eating and visits, etc.). Although these measures were taken in good faith, they prioritized the protection of physical health at all costs, without taking into account other aspects as mental health and quality of life for nursing home residents. These protective measures helped minimize the spread of the disease in nursing homes but imposed long periods of social isolation for the residents, which could have entailed a detrimental impact on their mental health [21]. This detrimental impact on mental health could, in turn, elevate the risk of other kinds of physical syndromes [38]. Indeed, preliminary studies showed that, in the US, nursing homes that applied more stringent protective measures suffered less infections from COVID-19 but had higher levels than in preceding years of non-COVID-19 related deaths during the first lockdown [11]. Thus, investigating how the first lockdown impacted residents’ wellbeing and what protected against a decline in quality of life is important to produce guidelines for possible future pandemics. Identifying the risk factors would allow to produce more balanced protective measures, in order to protect physically as well as psychologically this at-risk population. In addition, relatives of residents could also have been impacted by the protective measures in nursing homes. Since they could not see their residents during the lockdown, this could also have detrimental impact on their wellbeing and quality of life. Thus, in this rapid review, we aimed at investigating the literature on the impact of the COVID-19 protective measures on the wellbeing, quality of life and physical health of nursing home residents and their relatives. To this aim, we devised four questions that structured our literature search. Studies we selected in this rapid review were all related to at least one of these questions.

### Question 1A

What has been the impact of the measures to protect against COVID-19 on the physical and psychological health, quality of life and end of life support for nursing homes residents?

### Question 1B

Which interventions prevented/reduced the impact of the COVID-19 protective measures on the physical and psychological health, quality of life and end of life support of nursing homes residents?

### Question 1 C

What has been the impact of the protective measures against COVID-19 in nursing homes on the physical and psychological health and quality of life for close relatives of nursing homes residents?

### Question 1D

Which interventions prevented/reduced the impact of the protective measures against COVID-19 on the physical and psychological health, and quality of life for close relatives of nursing homes residents?

## Method

This rapid review followed the Cochrane Reviews Methods Group guidelines [20]. A rapid review is a form of literature synthesis that omits certain aspects of a systematic review in order to quickly produce evidence for decision makers. In particular, no formal evaluation of the quality of included studies is performed but included studies must have been published in a peer-reviewed journal. Nonetheless, a rapid review has been deemed the method of choice in order to inform stakeholders in a timely manner on the impact of the COVID-19 protective measures on the physical and mental health of residents in nursing homes and their relatives. The protocol for this rapid review was registered on Prospero (*reg.no.* CRD42022321398). Report of the rapid review follow the PRISMA statement for reporting systematic review [41].

### Search strategy and inclusion criteria

Systematic search was carried out on the 28th of March 2022 on the databases PubMed, PsycINFO and Embase.com. Regarding the outcomes, the search equations included a combination of terms related to “mental health” (e.g., “anxiety”, “wellbeing”), “physical health” (e.g., “pain”, “cognitive decline”, “physical autonomy”), and “quality of life” (e.g., “Wellbeing”). We also included terms relating to nursing homes (e.g., “long-term care”, “living facility for older people), nursing home residents (e.g., “older people”), and relatives of residents (e.g., “close relative”, “visitors”). Finally, the last set of keywords included words linked to restriction measures against the COVID-19 pandemic (e.g., “visit ban”, “containment strategy”, “COVID-19 restrictions”). Search equations can be found in the supplementary material. Since we were investigating the specific impact of COVID-19 protective measures on the mental and physical health of nursing home residents and their relatives, we restricted the search to the years 2020 and onward, and only included studies that specifically investigated the COVID-19 pandemic. The list of studies found in our systematic search were added to Rayyan, a management program aimed at facilitating systematic reviews [39].

To be included in the rapid review, the studies had to be written in French or English and published in a peer-review journal. We included quantitative as well as qualitative studies. Intervention studies were included even when no control groups were present, as long as the intervention was aimed at one of our populations of interest (residents or relatives). Systematic reviews were included if they evaluated one of our outcomes of interest. Opinion pieces, conference abstracts and editorials were excluded.

Titles and abstracts were screened independently by two researchers. In case of conflicts, a third researcher resolved conflicts. The next phase consisted in a full-text screening. This was done by five researchers. In case of doubts on the inclusion of a paper, the final decision was taken collegially by the five researchers. Finally, data extraction was performed by the same five researchers.

## Results

**Fig. 1:**
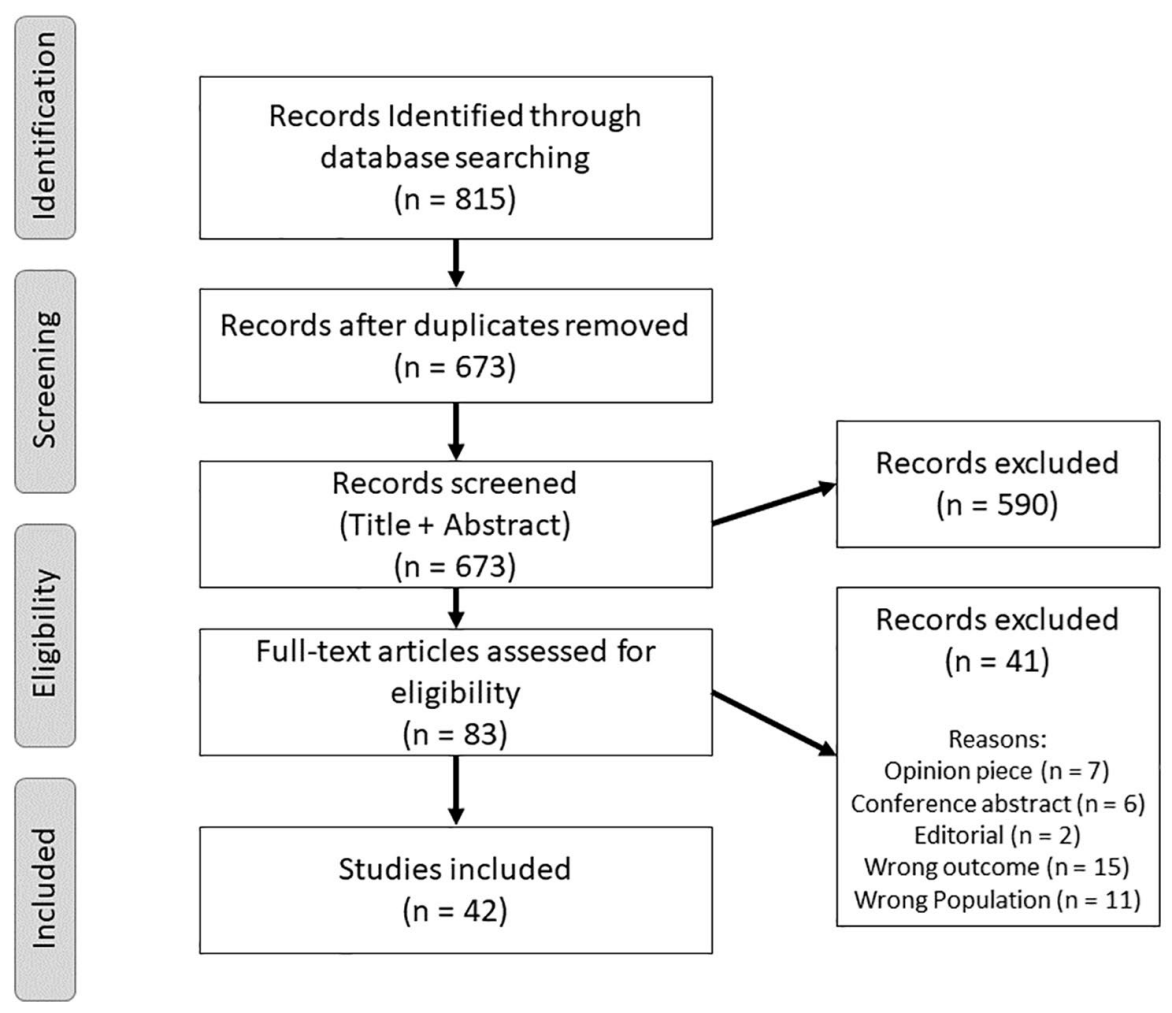
PRISMA flowchart of study selection

The databases search identified 815 papers. After screening, 42 papers were included in this rapid review (see Fig. 1). Twenty-height of these papers related to question 1 A, 4 to question 1B, 10 to question 1 C and none to question 1D. No systematic review of interest was identified during the screening process. For the ease of presentation, we will first present papers related to question 1 A, then 1B and finally 1 C. List of included studies can be found in Table 1.

**Table 1:** List of included studies, with the country of origin, general method, participants sample, number of participants, outcomes of interest, scales used (if any), and overview of the results

Primary study First author (Year)	Country	Design	Population	Sample	Outcome(s)	Scale(s) used (if any)	Results Overview
Aguilar et al. (2021)	USA	Longitudinal 3 time point, 6 weeks apart	NH resident	46	Anxiety, Depression	Geriatric Depression Scale, Geriatric Anxiety Inventory	No change on depression score, Lower anxiety score from T0 to T1, and then stay stable
Arpacioglu et al. (2021)	Turkey	Cross-sectional	NH resident & Older people living in autonomy	133 (66 NH residents, 67 auto.)	Depression, Anxiety, Stress	DASS-21, Satisfaction with life Scale	Stress, anxiety, depression higher in 80 + years people compared to 80- people. Higher depression, anxiety, and fear of death for NH residents compared to people living in autonomy
Ayalon et al. (2021)	Israel	Qualitative	NH residents	24	Experience of Lockdown		Residents reported a decrease in their overall wellbeing and high levels of depression. The loss of autonomy due to protection measure was also detrimental to their wellbeing.
Backhaus et al. (2021)	Netherlands	Qualitative	NH residents	64	Family visitation, Wellbeing		Five months after the end of the first lockdown, rates of visits were still lower than before the pandemic. Residents reported heightened wellbeing when relatives could visit them.
Borg et al. (2021)	France	Cross-sectional	Relatives	159	Anxiety, Depression, Caregiver Burden	General Anxiety Disorder Scale-7, Center for Epidemiologic Studies - Depression Scale, Zarit Burden Interview	Relatives of older people living in nursing home had heightened levels of depressive symptoms, anxiety, and sleep trouble than relatives who lived with their close elderly people.
Chen et al. (2021)	China	Intervention	NH residents with mild CI^1^ 2 groups: Control and Intervention	62	Depression, Physical Functioning	Geriatric Depression Scale	Positive impact of a physical exercise program on depression symptoms, quality of life and physical functions in NH residents with mild cognitive impairment
Chirico et al. (2022)	Italia	Qualitative	Relatives	26	Experience of Lockdown		COVID-19 restrictions had detrimental impact on relatives and their residents. Although videoconference tools were useful to alleviate that, there was limitations such as the capacity of residents to use these tools.
Curran et al. (2022)	Australia	Longitudinal 3 time points: before, during and just after the lockdown	NH resident	91	neuropsychiatric symptoms	NPI-NH	No impact of lockdown on neuropsychiatric symptoms prevalence
Danilovich et al. (2020)	USA	Secondary data analysis	NH residents	166	Weight change		Significant weight loss during the lockdown
Dupuis-Blanchard et al. (2021)	Canada	Qualitative	Relatives	17	Experience of Lockdown		Relatives reported that they had difficulties regarding communication with NH staff. They were sometimes in the dark regarding the actual restrictions put in place. In addition, they reported the detrimental impact the restriction had on the wellbeing of their residents.
Egeljic-Mihailovic et al. (2021)	Serbia	Cross-sectional	NH resident & Older people living in autonomy	299 (110 NH residents; 189 auto.)	Depression, social participation	Geriatric Depression Scale, Maastricht Social Participation Profile	Higher levels of depression in NH residents compared to people living in autonomy. Higher levels of depression of people living in urban areas compared to rural areas. Negative correlation between GDS and social participation
El Haj et al. (2020)	France	Cross-sectional	NH resident with CI^1^	58	Depression, Anxiety	Hospital Anxiety and Depression Scale	Anxiety and depression levels were higher during lockdown than previously
El Haj et al. (2021)	France	Cross-sectional	NH resident with CI^1^	72	Depression	Home-made Scale	Higher depression levels during lockdown than previously
Fogelson et al. (2021)	USA	Intervention	NH residents	18	Loneliness, Depression	Global Deterioration Scale, Geriatric Depression Scale, UCLA Loneliness Scale	Robotic pets decreased depression symptoms and feeling of loneliness in NH residents
Hindmarch et al. (2021)	Canada	Qualitative	Relatives	70	Role of Family Caregivers		Relatives reported wanting to have always access in some way to the NH, even though they would need to use stricter protection measures (distance, mask, etc.). Videoconference tools were not adapted to all residents.
Hoel et al. (2022)	Germany	Mixed	NH direction	417	Social Health, videoconference tool usage		Although videoconference tools were used to minimize social isolation of residents, there was several limiting factors to their use. Resident must have the cognitive capacity sot use them, must have learned to use them and must have support to help them use these tools.
Huber et al. (2022)	Switzerland	Cross-sectional	NH residents	828	Loneliness, satisfaction with life	Home-made scales	Heightened feelings of solitude during the lockdown
Kaelen et al. (2021)	Belgium	Qualitative	NH residents	56	Mental Health, Psychosocial Needs		The lockdown had detrimental effect on residents’ wellbeing. Psychosocial factors must be considered as potential source of suffering and must be evaluated via specific diagnostic tools
Koopmans et al. (2021)	Netherlands	Qualitative	NH staff & Residents’ relatives	72 staff, 73 relatives	Monitoring reopening		Lockdown lifting had substantial beneficial impact
Leontowitsc et al. (2021)	Germany	Qualitative	NH residents	22	Experience of Lockdown		Lack of social contact was difficult for the residents. Several participants reported that protecting against COVID-19 was not their top one priority; contact with family and overall wellbeing was more important.
Levere et al. (2021)	USA	Cross-sectional	NH residents	29,097	Weight loss, mental health, Cognitive functioning	Patient Health Questionnaire	Higher depression symptoms during lockdown than previously, significant cognitive decline during the first phase of the lockdown.
McArthur et al. (2021)	Canada	Longitudinal 3 time points Before, during and after the lockdown	NH residents	765	Depression, Behavioral problems	InterRAI	No impact of lockdown on rates of depression, delirium, or behavioral problems
Monin et al. (2020)	USA	Cross-sectional	Relatives	161	Mood, method of communication with Residents	Home-made Scale	Higher frequency of telephone between relatives and residents resulted in lower levels of negative emotions. Keeping contact with their residents was worrying for the relatives.
Murphy et al. (2022)	Ireland	Qualitative	NH residents	10	Experience of Lockdown		Residents felt that their physical health should not be protected at all costs and that social and cognitive stimulation must be taken into balance to design protection measure.
Nair et al. (2021)	Malaysia	Cross-sectional	NH residents	224	Depression, Anxiety, Social Support	Geriatric Depression Scale, Beck’s Anxiety Inventory, Multidimensional Scale of Perceived Social Support	Very high prevalence of depression symptoms during lockdown, high prevalence of anxiety symptoms. Lower levels of social support compared to pre-pandemic levels.
Nash et al. (2021)	USA	Qualitative	Relatives	518	Experience of Lockdown		Relatives reported that they suffered due to seeing their relative decline rapidly due to the lockdown. They treated isolation as inhumane, and reported that the lack of surveillance from the staff was the main reason they worried.
NG et al. (2020)	Singapore	Qualitative	NH residents	17	Physical activity, Experience of lockdown		Barriers to physical activity: closure of exercise facility, cancellation of group PA, and lack of equipment.
Noten et al. (2022)	Netherlands	Qualitative	Residents & Relatives	63	Experience of Lockdown		Relatives and residents described the lockdown as a difficult time, due to the social isolation induced by the protection measures. Although both groups understood why they were put in place, they still found isolation measure very harsh.
O’Caoimh et al. (2020)	Ireland	Cross-sectional	Relatives	225	Loneliness, Wellbeing, Quality of Life	UCLA Brief Loneliness Scale, WHO-5 Wellbeing Index, Adult Care QoL Questionnaire	Support for the relatives as caregivers was poor during the lockdown, which impacted the wellbeing of relatives.
Paananen et al. (2021)	Finland	Qualitative	Relatives	41	Experience of Lockdown		Relatives reported negative impact of the protection measure on their relationship with their resident. Many reported physical as well as cognitive deterioration during the lockdown.
Pereiro et al. (2021)	Spain	Longitudinal 4 time points: 3 during lockdown and one after	NH residents	98	Depression, Social Contact	Geriatric Depression Scale	Social contact attenuates the detrimental impact of lockdown on depression symptoms
Perez-Rodriguez et al. (2021)	Spain	Cross-sectional	NH residents	435	Nutrition, Cognitive functioning	Barthel Index, Global Deterioration Scale	Worsening of the functional, cognitive, emotional and nutritional status of NH residents. No impact whether they contracted COVID-19 or not on these outcomes.
Pinazo-Hernandis et al. (2022)	Spain	Intervention	NH residents	34	Mood, Depression, Anxiety, Loneliness	Gierveld’s Loneliness Scale, Goldberg’s Depression and Anxiety Scale	Positive impact of a reminiscence program on feeling of loneliness, depression & anxiety symptoms, and positive & negative affects
Plangger et al. (2022)	Austria	Longitudinal 4 time points: 2 before lockdown and 2 after	NH residents	48	Depression, Anxiety, Quality of Life	Geriatric Depression Scale, Beck’s Anxiety Inventory	Increase in depression and anxiety symptoms during the lockdown, and then decrease after reopening.
Prins et al. (2021)	Netherlands	Cross-sectional	Relatives	958	Worries, Loneliness	Home-made Scales	Relatives that visited more than once a week had higher levels of worries than other relatives. This was modulated by the resilience of the relatives, with more resilient relatives having less worries.
Savci et al. (2021)	Turkey	Cross-sectional	NH residents	103	Loneliness, Quality of Life, fear of COVID-19	Loneliness Scale for Elderly, WHOQOL-BREF	Fear of COVID-19 was heightened for people with high levels of social relation and higher quality of life.
Sizoo et al. (2022)	Netherlands	Longitudinal 4 time points: Once a month after the lockdown	NH residents	252	Depression, Anxiety, Agitation	Neuropsychiatric Inventory Questionnaire	Higher levels of depression and agitation at the beginning of the lockdown than previously, but monotonic decrease linked to reopening.
Van Der Roast et al. (2020)	Netherlands	Cross-sectional	NH residents	193	Mood, Behavioral Problems	Mental Health Inventory − 5	High levels of depression, loneliness and behavioral problems 6 to 10 weeks after the beginning of lockdown than before. Residents without CI^1^ more impacted than residents with CI^1^
Van Dyck et al. (2020)	USA	Intervention	NH residents	30	wellbeing	Home-made Scale	Positive impact of a telephone outreach program in which medical students phoned NH residents once a week.
Verbeek et al. (2020)	Netherlands	Qualitative	NH staff	30	Reopening, mental health		There was variation in how NH implemented protection measures. This was mainly due to difference in resource that could be applied. Nonetheless, all NH welcomed the reopening following the lockdown.
Wammes et al. (2020)	Netherlands	Qualitative	Relatives	1997	Experience of Lockdown		Relatives perceived that loneliness, sadness and loss of quality of life would be the main impact of the protection measures. Nonetheless, the majority of relatives found that the measures were necessary.
Zamora et al. (2022)	Spain	Longitudinal	NH residents	215	Depression, Anxiety, Functional Ambulation	Geriatric Depression Scale, Hospital Anxiety and Depression Scale, Barthel Index	Depression: no difference whether contracted COVID-19 or not, but higher overall prevalence than previously. Anxiety: COVID-19 patients > not infected, overall levels higher than before the lockdown Sleep: no difference between infected and non-infected, but overall high levels of sleep problems.

^1^CI: Cognitive Impairment

Studies found in our search mostly investigated the impact of the first lockdown induced by the first wave of COVID-19 which lasted from March to June 2020. Most of the studies were produced in European and North American countries (25 studies from Europe, 10 from North America). The rest of included studies came from the Middle East countries (3 studies), East-Asian countries (3 studies) and one study from Australia. In the included quantitative studies, sample size ranged from 36 in an intervention study to 29’097 in another study using data from healthcare providers. In Europe and in North America, the lockdown started in March 2020 and protective measures were relaxed at the end of the first wave, during May and June 2020, depending on the country. Some country kept stringent protective measures throughout 2020, as Turkey for example. In the results’ presentation below, the “lockdown” thus refers to protective measures put in place during the March to June 2020 period. If studies presented refers to another period of the pandemic, then it will be formally expressed.

### Question 1 A: What has been the impact of protective measures on the physical and psychological health, quality of life and end of life support for nursing home residents?

We found twenty quantitative studies and eight qualitative studies related to question 1 A. All eight qualitative papers related to the overall subjective experience of residents during visit bans. Quantitative papers were divided in three main categories, depending on their outcomes of interest. Fourteen papers investigated depression and anxiety, three investigated loneliness and overall quality of life, and four investigated medical aspects, like physical activities, cognitive decline and nutrition. In the following section, we will present studies using quantitative methods. Qualitative studies will be presented in a separate section.

#### Impact of protective measures on depression and anxiety of residents

In the fourteen papers investigating depression and anxiety as an outcome, five used a longitudinal methodology. Aguilar and colleagues (2021) [1] found that levels of depression and anxiety were kept mostly stable during the two months following the first lockdown. The McArthur and colleagues study (2021) [30] used InteRAI data, which were collected monthly before and during the first lockdown at the beginning of 2020, to evaluate whether levels of depression and anxiety changed during this period. They found no impact of the first lockdown on their measures.

Beside these two studies, the other papers presented in this section all found a detrimental impact of protective measures on levels of depression and anxiety of residents. Arpacioğlu and colleagues (2021) [2] found that older people living in nursing homes had higher levels of depression and anxiety than older people living in autonomy. Their results also showed that overall life satisfaction of residents in nursing home was higher when relatives came to visit more than 2 h per week. The difference between nursing home residents and older people living in autonomy was corroborated by the Egeljić-Mihailović and colleagues’ study (2022) [16]. They also found that residents had higher levels of depression than autonomous older people. In addition, their data showed that older people living in urban areas had higher levels of depression than older people living in rural areas.

One study compared overall levels of depression pre-pandemic found in the literature with levels of depression measured just after the first lockdown [33]. They found that overall levels were higher than pre-pandemic levels, and residents with higher social support had lower levels of depression. Leontowitsch and colleagues (2021) [28] compared residents on their levels of depression before and after the first lockdown. They found that residents had higher levels of depression after it than before. In addition, this study used a mixed method where residents were also interviewed. Analysis of the interviews showed that an absence of group activities and changes in their routine negatively impacted their overall wellbeing.

Two longitudinal studies were able to compare directly levels of depression and anxiety before and after the first lockdown. Pereiro and colleagues (2021) [42] showed that depression levels were higher after the first lockdown compared to before the first lockdown. However, the effect disappeared when social contact frequency was taken into account. The authors thus concluded that social contact could shield against the possible detrimental impact of protective measures. This was corroborated by the study from Plangger and colleagues (2022) [45]. They found that levels of anxiety and depression increased during the first lockdown, and then decreased afterward, which was interpreted as evidence for the detrimental impact of lockdown on these outcomes.

Two other longitudinal studies investigated levels of depression and anxiety during the months following the first lockdown (May-September 2020). Sizoo and colleagues (2022) [48] found that levels of depression and frequency of agitations gradually decreased following the end of the first lockdown, but overall levels of both outcomes were still heightened compared to levels found in the literature for this population. Cortès Zamora and colleagues (2022) [10] also found that levels of depression were twice as high as the pre-pandemic mean. Patients that contracted COVID-19 had higher levels of depression than patients that did not contract it. Finally, they found the overall decrease in functional mobility after three months of lockdown would normally be found after one year.

One last study was able to compare levels of depression during the first lockdown with the same measure from previous years [29]. The authors found that depression levels were higher during lockdown than preceding years but started to decrease when nursing homes started reopening. They also found that there was more cognitive deterioration during the first lockdown than the preceding years.

Studies presented above investigated older people without cognitive impairment. Now we will present three studies that were directly aimed at investigating anxiety and depression in nursing home resident diagnosed with cognitive impairment. El haj and colleagues (2020) [17] investigated perceived levels of depression and anxiety in residents with cognitive impairment compared to healthy residents. They found that residents with cognitive impairment self-reported higher levels of anxiety and depression after the first lockdown than before it. The follow-up to this study [18] used the same methodology, but this time levels of depression and anxiety were rated by formal caregivers of residents. The results showed that caregivers reported that residents had higher levels of depression after the first lockdown compared to before it. However, level of cognitive impairment was not correlated to perceived depression in residents. Finally, Hoel and colleagues (2022) [23] use a mix of qualitative and quantitative data to investigate levels of depression, anxiety and social participation in residents with cognitive impairment during the first lockdown. Formal caregivers reported heightened levels of depression and anxiety during lockdown compared to before it. Qualitative analysis of semi-structured interviews showed that formal caregivers found new communication technologies were very helpful to maintain social support for the residents.

#### Impact of protective measures on loneliness and quality of life of residents

Three studies investigated whether the first lockdown had an impact on loneliness and overall quality of life. Savci and colleagues (2021) [47] used retro-active questionnaires to evaluate the quality of life of nursing home resident in Turkey. Results showed no difference at the moment of measure (November/December 2020) and retro-active judgement of their quality of life during the first lockdown. Another study that compared levels of loneliness between residents with and without cognitive impairment [50]. They found overall high levels of loneliness compared to what was found in the literature. In addition, resident without cognitive impairment had higher levels of loneliness than participant with cognitive impairment. Finally, the study from Huber and colleagues (2022) [24] found that, when participants had to judge their levels of loneliness at the time of reopening (May 2020), and retro-actively compare it with levels before the pandemic, the residents felt higher levels of loneliness at the time of reopening. Nursing home residents also had higher level of loneliness compared to older people living in autonomy.

#### Impact of protective measures on physical function, cognitive decline and nutrition of residents

Fours studies used a quantitative approach to evaluate the impact of the first lockdown on physical functions, cognitive decline and nutrition. Perez-Rodriguez and colleagues (2021) [43] evaluated how food-intake, ambulation and levels of depression were impacted by the first lockdown. Their results showed a worsening in nutritional assessments in more than 90% of residents. In addition, there was a high prevalence of depressive symptoms, as well as a degradation of ambulation capacity for the residents. Interestingly, there was no difference whether residents had contracted COVID-19 or not. Danilovich and colleagues (2020) [13] investigated weight change from December 2019 to April 2020. They found that there was a significant weight loss between March and April, which was attributed to visits ban and the impossibility to eat in groups. Ng and colleagues (2020) [35] used qualitative methods to evaluate whether residents kept physically active during lockdown, and what factors influenced possible change in physical activity. Residents reported being less physically active during the lockdown.

Finally, Curran and colleagues (2022) [12] investigated whether prevalence of neuropsychiatric symptoms changed during the lockdown in Australia. There, a first small wave happened in March-April 2020, and a second, more important wave, happened between July and September 2020. The authors measured symptoms before the first wave, just after the reopening following the first wave and just after the reopening following the second wave. Results showed no difference on the prevalence of neuropsychiatric symptoms between the three time-points.

#### Qualitative studies investigating the impact of protectives measures on the wellbeing and quality of life of nursing home residents

Our search found eight qualitative studies investigating the experience of nursing home residents during the lockdown induced by the first wave of COVID-19. Ayalon and colleagues (2021) [3] interviewed residents of nursing homes in Israel on how they felt during the lockdown. Residents reported that their wellbeing and mental health deteriorated during the lockdown. Although most of the residents understood why the measures were put in place, they nonetheless felt that they lost control over they own life due to these, and this was detrimental to their overall wellbeing. Finally, residents reported that they welcomed reopening, because they could again be directly in contact with their close ones.

The impact of the loss of autonomy on wellbeing due to the protective measures was also reported in the study from Kaelen and colleagues (2021) [26]. This study, which relied on semi-structured interviews, also investigated how residents felt during the first lockdown. Residents reported a loss of self-determination and autonomy due to the protective measures. This was compounded by the absence of social life, which exacerbated negative feelings. Indeed, many residents reported that the absence of direct contact with relatives, the absence of social gathering and other measures of that kind impacted directly their wellbeing. In addition, residents felt patronized and infantilized by the measures and felt that they were unjust.

Backhaus et al. (2021) [4] investigated how residents and their relatives experienced the reopening following the first lockdown. Overall, both residents and relatives welcomed the reopening. They reported that seeing directly their close ones was better than via videoconference tools. In addition, the authors found that, five months after the reopening, visits did not reach pre-pandemic levels.

Murphy and colleagues (2022) [32] aimed at investigating the experience of residents that moved from a multi-beds room to an individual room when the lockdown was in place. Residents reported that moving to individual room had a positive impact, because residents could have more personal control over their direct environment. This beneficial impact of the move was enhanced by the possibility to go outside for walks. Residents reported that visits ban was difficult, as it increased their loneliness and isolation. Distanced visit (like visit at the window) or videoconference helped alleviate the negative feelings, but they did not replace direct, physical contacts.

Paananen and colleagues (2021) [40] interviewed relatives on the perceived impact of the protective measures on their wellbeing and the wellbeing of residents in Finland. Almost all relatives reported a negative impact of the protective measures on both their and their resident wellbeing. Several relatives saw a rapid cognitive and physical decline of the residents that they attributed to the protective measures (absence of social contact, isolation). Finally, relatives reported feeling of anxiety and sadness for them and their residents.

The last two studies included in our search investigated qualitatively the reopening following the first lockdown. First, Verbeek and colleagues (2020) [53] investigated reopening from the residents’ point of view. Analysis of interviews showed that residents welcomed the reopening. They also reported that in-person meeting was better than other compensatory solutions (videoconference, “window” visits, etc.). Of interest, the authors showed that there was no new COVID-19 infection in the three weeks following reopening in the nursing homes participating in this study. The direct follow-up to this study [27] showed that most visitors of nursing homes readily followed protectives measures (masks, handwashing, hand gel, etc.). Residents were also in a better mood after reopening, which was likely due to being allowed to see their relatives.

### Question 1B: Which interventions prevented/reduced the impact of the COVID-19 protective measures on the physical and psychological health, quality of life and end of life support of nursing homes residents?

There were four intervention studies aimed at reducing the impact of protective measures on physical functions and mental health. The Pinazo-Hernandis and colleagues’ study (2022) [44] examined the effect of a reminiscence program on levels of depression, anxiety, feeling of loneliness, and negative and positive affect. A reminiscence program is a kind of psychological intervention, where participants recall past events and important people from one’s life. This reminiscence is used to review experiences of older people, promote positive feelings, and give meaning to past and present experiences of participants [55]. It has been shown that these kinds of interventions have a positive impact on depression, anxiety, and psychological wellbeing for older adults living in nursing homes [25]. The control group showed a monotonic increase in all outcomes (levels of depression, anxiety, feeling of loneliness and negative affect), whereas the intervention group showed a decrease in levels of anxiety. Depression levels and negative affect kept stable for the intervention group. Thus, the authors concluded that the reminiscence program had an overall beneficial impact on residents’ level of anxiety and protected against an increase in depression levels and negative affect.

The intervention study from Van Dyck and colleagues (2020) [51] investigated whether a telephone outreach program would improve wellbeing of residents in nursing homes. Medical students were enrolled to call a set of nursing home residents once a week at regular schedule. The authors only evaluated the impact of this program qualitatively. Students and residents reported positive impact of this outreach program on the overall wellbeing of residents. Students said that they felt helpful to the residents, and residents reported that the feeling of connection with the students was important for them.

The Chen and colleagues’ study (2021) [8] investigated the effect of a physical exercise program, the OTAGO program, on physical functions, mental health, and quality of life of residents. The OTAGO program consists of a 30-minutes set of physical exercises specifically aimed at improving balance and muscle-strength for older people [6]. Participants in the intervention group participated in three intervention sessions a week during twelve weeks. Results showed that the intervention had positive impact on residents. Participants in the intervention group showed a monotonic improvement in mental health, quality of life and physical functions outcomes. Inversely, participants in the control group deteriorated on measures of mental health, physical functions and quality of life.

Finally, Fogelson and colleagues (2021) [19] investigated whether giving robotic pets to residents with mild to severe cognitive impairment would impact their levels of loneliness and depression. The researcher used a mixed-method design. Quantitative data showed that loneliness decreased after the beginning of the intervention, and then kept stable during the follow-up measures. Depression levels decreased monotonically in the intervention group. Analysis of qualitative data corroborated this pattern of findings. Residents and professional caregivers viewed the robotic pets positively.

### Question 1 C: What has been the impact of the protective measures against COVID-19 in nursing homes on the physical and psychological health and quality of life for close relatives of nursing homes residents?

Our search found eleven studies that investigated the psychological impact of the protective measures against COVID-19 in nursing home on the relatives of residents. Four studies used a quantitative approach, whereas seven investigated the experience of relatives with the protective measures via qualitative designs. First, we will present the four quantitative studies, then, in another section, the qualitative studies.

#### Quantitative studies investigating the impact of protective measures on psychological health and overall quality of life of relatives

Borg and colleagues (2021) [5] investigated levels of depression, anxiety, stress, and caregivers’ burden of relatives after the first lockdown. Participants were pooled in two groups, depending on whether the older were living with the relatives or in a nursing home. Results showed that relatives of older people living in nursing home had heightened levels of depressive symptoms, anxiety, and sleep trouble than relatives who lived with their close older people. In the same vein, O’Caoimh and colleagues (2020) [37] investigated how the lockdown and the amount of social support impacted quality of life of relatives of nursing homes’ residents with cognitive impairment. They found that the overall quality of life of relatives depended on the perceived support received from nursing home staff, with lower perceived support linked to lower quality of life. The quality of life of relatives was also influenced by the degree of cognitive impairment of the residents. Relatives of residents with more pronounced cognitive impairment had lower quality of life than relatives of residents with less pronounced cognitive impairment.

Prins and colleagues (2021) [46] wanted to investigate whether the impact of the protective measures on relatives was linked to relatives’ resilience and pre-pandemic visit frequency. The authors defined resilience as a personal characteristic which serves as a protective barrier against developing certain psychological problems. Results showed that relatives that visited more often before the pandemic had more worries than relatives that visited less often. In addition, relatives with higher levels of resilience were less impacted than relatives with lower levels of resilience.

Finally, Monin and colleagues (2021) [31] investigated how relatives kept contact with their residents, and how it impacted the negative and positive affect of relatives as well as the perceived positive and negative affect of residents from relatives’ point of view. Results showed that relatives that phoned more often their residents reported fewer negative emotions, compared to relatives that phoned less often. In addition, residents that received e-mails from close ones more often were perceived as having more positive affect than residents that received less e-mails. Conversely, a greater frequency of letters was associated with more negative affect in relatives as well as in residents.

#### Qualitative studies exploring how relatives experienced the protective measures against COVID-19 in nursing homes

Six qualitative studies investigated how relatives experienced the protectives measures. Chirico and colleagues (2022) [9] explored the subjective experience of relatives of nursing home’ residents regarding protective measures, how the measures impacted their lives and what relatives did to alleviate this impact. Relatives reported a worsening of their wellbeing and high levels of stress. This was, in part, due to relatives seeing the rapid deterioration of their residents, physically and psychologically. Relatives also reported feeling being left out by formal caregivers and attributed their heightened levels of stress to the absence of social contact. Relatives welcomed the use of videoconference tool to keep some contact with residents, but they also reported that it could not completely replace direct physical contact. In addition, they also reported that videoconference tool was not appropriate for all residents. Hearing impairment, lack of knowledge about computer and lack of support from staff hindered the use of videoconference tools for residents. Hindmarch and colleagues (2021) [22] explored what was important for relatives regarding their residents during the first lockdown. Relatives reported that not being allowed to visit their residents was difficult. They wanted to have access to nursing homes during the lockdown, because many relatives also have a caregiver role. In this aim, relatives also agreed to use other kind of protective methods (masks, hygiene caps, hand gels, etc.), as long as they were allowed to visit the residents. Relatives also reported that, although communication technologies were useful to keep contact with the residents, they did not replace in-person meetings.

Nash and colleagues (2021) [34] also explored how relatives experienced measures against COVID-19 in nursing homes in the USA. Relatives reported being worried about their residents who were in isolation. Several relatives noted a rapid decline of the residents, and some wanted to keep being able to see their residents, despite the risk of infection. Indeed, these relatives found that it was more important to socially connect with residents than to protect them at all costs from infection. Isolation was hard for the residents, especially for cognitively impaired residents, because they did not understand why the measures were put in place. The study argues for a special status for relatives that help giving care to residents, as these cares are important for the overall wellbeing of residents. Noten and colleagues (2021) [36] aimed at the same goal as the previous study, but in Flanders (Belgium). Relatives reported that protective measures impacted the social life of residents and relatives. Although communication technologies helped alleviate this impact, they did not replace in person visits. In addition, the relatives reported that communication tools like videoconference program were not suitable for every resident. Some had problem to use these technologies, especially residents with cognitive impairment. All relatives welcomed reopening, even partial. However, for some relatives, meeting their residents after not seeing them for two months was hard, because some residents did not recognize their relatives (due to wearing masks and cognitive decline experienced during the lockdown). Finally, relatives argued that social and psychological life should also be promoted during a pandemic, and not focus only on the physical risk of infections.

Wammes and colleagues (2020) [54] explored the subjective impact of protective measures on life satisfaction and its link to the frequency of visits in the Netherlands. Relatives which visited their resident more frequently reported being more satisfied with life than relatives that visited less frequently. In addition, relatives argued for the reconnaissance of a specific role for family caregivers, as they have an important role in the overall care given to residents. Finally, relatives reported fearing that protective measures would impact negatively the wellbeing of residents.

The final studies selected by our search methodology interviewed relatives about their perception of the protective measures in Canada and the problems they elicited [15]. Relatives reported having difficulties to communicate with nursing homes staff. They reported that it was somewhat difficult to have news from the residents and to be updated on the ongoing measures, which changed several times during the first wave COVID-19 in 2020. The authors found that the autonomy of residents had an impact on the wellbeing of relatives: more autonomous residents could more easily give news to their relatives, which in turn impacted positively the wellbeing of relatives. Relatives also reported an increasing feeling of distress as the lockdown continued, which they linked to the absence of direct contact with the residents.

## Discussion

The aim of this rapid review was to investigate what has been published on the impact of protective measures against COVID-19 in nursing homes on the wellbeing, quality of life, psychological health and physical health of residents and their relatives. Our systematic search found 42 papers that related to our questions of interest. Regarding residents, most of the papers reviewed here corroborated the hypothesis that mental health as well as physical health of nursing homes residents deteriorated during the first lockdown. First, several studies showed that residents had higher levels of depression and anxiety after the first lockdown than before it [28, 42, 45]. In addition, residents had higher levels of depression after the first lockdown compared to levels found in the literature for this population [10, 33, 48]. Several other studies showed that depression and anxiety levels decreased following the reopening [29, 45]. Taken together, these results show that the lockdown period had a detrimental impact on the wellbeing, psychological health and physical health of residents.

However, these studies do not inform whether the impact of the lockdown was specific to nursing home residents or extends to the general population. Therefore, some studies compared nursing home residents with older people living in autonomy [2, 16]. In both studies, the authors found that nursing home residents had higher levels of depression and anxiety than older people living in autonomy. This shows that nursing home residents were more impacted by the protective measures during the lockdown than residents living in autonomy. The lockdown also impacted feelings of loneliness for residents. Two studies showed that residents had higher levels of loneliness just after the first lockdown than before it [50, 24]. Regarding food-intake, two studies found that residents lost significant weight during the first lockdown, and that more than 90% of residents had a worsening of their nutritional assessment [13, 43].

The detrimental impact of the lockdown period on nursing homes residents was likely due to the COVID-19 protective measures. In several qualitative studies residents reported the direct link between their perceived decrease in wellbeing and protectives measures (isolation, lack of social contact, lack of activities, etc.). For example, residents reported feelings of loneliness, heightened levels of depression, and anxiety [3, 26] and linked these experiences directly to the lack of social contact. They also reported feeling a loss of self-determination over their lives, which exacerbated their negative feelings.

Although it is assumed that residents suffered the most from the protective measures, because measures were more restrictive in nursing homes, relatives of residents also suffered from these measures. People that lived with their older relatives had less anxiety, depressive symptoms, and sleep troubles than relatives of nursing homes residents of the same age [5]. Overall wellbeing of relatives depended on the perceived support their residents were given by the staff [37]. In addition, a study insisted on a heightened burden for relatives, because they could not engage in the care they were giving before the pandemic. Indeed, one study found that relatives that visited more their residents before the pandemic suffered more from the lockdown than relatives who visited their residents less often [46]. Qualitative studies exploring how relatives experienced the protective measures in nursing homes also corroborate their impact on the overall wellbeing of relatives. In several studies, relatives reported being worried for their residents [9, 34] and felt that the protective measures would have a negative impact on the residents [40], which would in turn increase their worries.

### Protecting factors against the detrimental impact of protective measures on nursing home residents, and interventional solutions

We saw in the preceding section that protective measures in nursing homes negatively impacted the wellbeing and quality of life of residents and their relatives. However, the studies found in our rapid review also presented several avenues to alleviate this detrimental impact. The main preventive factor against the psychological impact of protective measure was social contact. Indeed, two studies showed a negative correlation between social contact and levels of depression and anxiety [16, 33]. Another study showed that the relation between the lockdown and higher levels of depression and anxiety disappeared when social contact frequency was added as a predictor [42]. The importance of social contact was also evidenced by studies that showed a decrease in levels of depression and anxiety when protective measures relaxed and residents were able to meet more directly with their relatives [10, 48]. The importance of direct contact was also largely reported in qualitative studies. Residents reported that the lack of contact with relatives or other residents was very difficult for them [3, 26, 32] and link the heightened levels of depression and anxiety to the absence of social contact.

To counteract the detrimental impact of the lack of social contact on residents, several authors proposed to use videoconference tools (tablets, smartphones). This allows residents and their relatives to keep contact and minimize social isolation. Although residents and relatives welcomed the use of videoconference tools, it had several intrinsic problems. First, not every resident is able to use these technologies. For example, residents with hearing impairment or cognitive impairment struggled with videoconference tools. In addition, residents and relatives preferred in-person visits to videoconference, although videoconference tools were considered a good temporary solution [3, 4, 23].

Several authors argued that totally isolating residents from the outside was not a good solution [34, 36]. These authors argued that some relatives should always be able to visit their residents, albeit with additional protective measures (masks, hygiene caps, full-body hygiene suits, protective glasses, etc.). There are three main reasons that support this conclusion: firstly, relatives that visit their residents often also have an important role as caregivers. Stopping these relatives to visit their residents would thus hinder the wellbeing of residents [22]. Secondly, allowing relatives in the nursing home would alleviate the most detrimental aspect of the protective measures: the lack of social contact. Finally, preliminary studies have shown that three weeks after reopening, there was no new COVID-19 infection [53] in nursing homes. Thus, it does not seem that allowing relatives back in nursing homes increases risks of infections. This is likely due to the relatives’ propension to readily follow other protective measures like masks and hand gel [27]. Although these are preliminary results and must be investigated further, it illustrates that letting relatives back in the nursing homes would not increase the risk to the residents while improving greatly the wellbeing of residents and relatives.

Regarding interventions that could help alleviate the detrimental impact of the protective measures, doing weekly physical exercise [8] and engaging in a reminiscence program [44] have been shown to have a positive impact on depression and anxiety levels. Part of the positive impact is likely due to the group activities, which let residents be in contact with other residents instead of being kept in their room, but part of it comes directly from the intervention. Having regular telephone talks with outsiders has also been shown to have a positive impact [51]. But, as we saw earlier, this cannot be put in place for every resident, as telephone can be difficult of residents with hearing impairment or cognitive impairment. Nonetheless, it is a cost-effective temporary solution that can help a sizeable portion of the nursing homes residents’ population. Interestingly, we found no studies that investigated intervention that could help alleviate the detrimental impact of the lockdown on relatives of residents. However, since it has been shown that relatives that talked with their residents more often had less worries than relatives that talked less often with residents, letting residents and relatives use videoconference tools to communicate should have a beneficial impact on relatives.

### Recommendations to minimize negative impact of protective measures on psychological health of residents

Several papers that were found in the literature search proposed recommendations on how to balance psychological health with the physical protection of nursing home residents from infections. Some of these studies were not included in our final set of included studies, because they did not follow our inclusion criteria. We nonetheless found useful to list recommendations made by these papers here, as they could help develop protective measures that balance mental and physical health in case of a future pandemic.

Dichter and colleagues (2020) [14] proposed a set of recommendations to minimize social isolation of residents. As we saw in the preceding section, videoconference tools can be used to help residents keeping contact with their relatives. Thus, staff should support residents with the use of tablets (or other similar device), because one of the barriers for resident to use these kinds of technologies rely on their knowledge of how they work. In addition, the authors argued that relatives should always be allowed to visit their residents (in compliance with safety protocols) and residents should be able to spend time outside. Regarding deterioration of mental health, nurses should be trained to be as much wary of psychological symptoms as physical symptoms and spend more time with the residents to alleviate social isolation. However, we acknowledge that this course of action is difficult to put in place at the moment in the current context of staffing shortages. In addition, it has been shown that pandemics also have an impact on the mental health of healthcare professionals [49]. This, in turn, would likely induce an increase in turn-over and a decrease in the quality of healthcare. Thus, protective measures should also not heighten the burden put on healthcare professionals. Regarding relatives, many qualitative studies investigating their experience during the first lockdown showed that the levels of worries they experienced depended on the perceived support given by nursing homes staff. Thus, at least one paper argues for assigning a reference staff to each family [52]. This reference staff would have the role of informing families on that state of the residents and updates on the protective measures (possibilities and time of visits, protective protocol, etc.).

## Limitations

Rapid reviews have inherent drawbacks, like the absence of formal quality evaluation of the included studies [20]. In addition, we limited our search to a subset of databases and only included articles in French and English. Furthermore, we did not search for grey literature (non-commercial or unpublished material, e.g., official institutional report, government report, etc.). Nonetheless, the fact that we only included peer-reviewed articles and our strict inclusion/exclusion criteria limited the possibility for bad quality articles inclusion. Another strength of this rapid review is that we included qualitative studies, which give important information on how people lived through the lockdown. Finally, the help of a professional librarian for the development of search equations also enhanced the quality of our bibliographical research.

## Conclusion

Our review of studies investigating the impact of the COVID-19 protective measures in nursing homes showed that these measures had detrimental impact on the wellbeing and quality of life of residents and their relatives. Although the measures helped protecting residents against infections, they also imposed psychological stress on people which had to live with these measures. Furthermore, recent studies linked more stringent protective measures to a heightened number of non-COVID-19 related deaths during the first lockdown. Since the role of nursing homes is to preserve the quality of life of their residents, it asks the question as to how they can fulfill this role while protecting residents from infections. We saw that the detrimental impact of the measures was mostly due to residents’ lack of social contact and loss of self-determination. Thus, measures that minimize social isolation and preserve self-determination should be preferred. Another aspect is that relatives of residents were also impacted negatively by the protective measures. Since relatives of nursing homes residents also have an important role in the caregiving of residents, they should not be considered as mere visitors, but as an integral part of the caregiving structure. Thus, in the case of a new pandemic similar to COVID-19, relatives should be considered as partner with the nursing homes and not be completely left-out. This would have the double effect of minimizing residents’ social isolation and relatives’ worries.

### Electronic supplementary material

Below is the link to the electronic supplementary material.

Supplementary Material 1

## Data Availability

All data generated or analyzed during this study are included in this published article.
