# Supplementary material for: The impact of protective measures against COVID-19 on the wellbeing of residents in nursing homes and their relatives: a rapid review
Source: BMC Geriatr. 2023 Oct 11;23:649. doi: 10.1186/s12877-023-04300-7 (PMC10568910; doi:10.1186/s12877-023-04300-7)
Supplement: Supplementary file 1 — Supplementary Material 1 [file 12877_2023_4300_MOESM1_ESM.docx]

# Supplementary Material

## Search Equations

## PubMed

(((COVID-19*[MeSH Terms]) OR (SARS-CoV-2[MeSH Terms]) OR ("coronavirus infections"[MeSH Terms]) OR (Pandemics[MeSH Terms]) OR (COVID-19*[Title/Abstract]) OR (SARS-CoV-2[Title/Abstract]) OR (Coronavirus Infections[Title/Abstract]) OR (Pandemics[Title/Abstract])) AND (("residential facilities"[MeSH Terms]) OR (residential facilities[Title/Abstract]) OR ("homes for the aged"[MeSH Terms]) OR ("nursing homes"[MeSH Terms]) OR (Nursing Homes[Title/Abstract]) OR (homes for the aged[Title/Abstract]) OR (resident*[Title/Abstract])) AND (("patient safety"[MeSH Terms]) OR ("patient safety"[Title/Abstract]) OR (Visitors to Patients[Title/Abstract]) OR ("visitors to patients"[MeSH Terms]) OR ("restrictive measures"[Title/Abstract]) OR ("visiting bans"[Title/Abstract]) OR ("COVID* measures"[Title/Abstract]) OR (lockdown[Title/Abstract]) OR (isolation[Title/Abstract]) OR ("social isolation"[MeSH Terms])) AND (("quality of life"[MeSH Terms]) OR (quality of life[Title/Abstract]) OR (well-being[Title/Abstract]) OR ("mental health"[MeSH Terms]) OR (mental health[Title/Abstract]) OR (acute stress[Title/Abstract]) OR (depression[MeSH Terms]) OR (depression[Title/Abstract]) OR (physical health[Title/Abstract]) OR (psychological health[Title/Abstract]) OR (end of life support[Title/Abstract]) OR (end of life support[MeSH Terms]) OR ("psychological distress"[MeSH Terms]) OR (psychological distress[Title/Abstract])))

## EMBASE

('coronavirus disease 2019'/exp OR 'severe acute respiratory syndrome coronavirus 2'/exp) AND ('quality of life'/exp OR 'wellbeing'/exp OR 'mental health'/exp OR 'acute stress'/exp OR 'depression'/exp OR 'covid-19 related psychological distress'/exp) AND ('quality of life'/exp OR 'wellbeing'/exp OR 'mental health'/exp OR 'acute stress'/exp OR 'depression'/exp OR 'covid-19 related psychological distress'/exp) AND ('nursing home'/exp OR 'resident'/exp OR 'home for the aged'/exp)

## PsycInfo

((Coronavirus/ or covid-19/ or severe acute respiratory syndrome/ or (severe acute respiratory syndrome or coronavirus or covid-19).af.) AND (nursing homes/ or residential care institutions/ or nursing home residents/ or long term care/ or (resident* or nursing homes or home* for the aged).af.) AND (patient safety/ or social isolation/ or quarantine/ or social exclusion/ or (patient safety or visitors to patients or restrictive measures or lockdown or social isolation or visiting bans or COVID measures).af.) AND (exp "Quality of Life"/ or well being/ or mental health/or "depression (emotion)"/ or (quality of life or mental health or depression or psychological distress or wellbeing or acute stress or physical health or psychological health or end of life support).af.))

## Web of Science (Core collection)

((COVID-19*) OR (SARS-CoV-2) OR ("coronavirus infections") OR (Pandemics) OR (COVID-19*) OR (SARS-CoV-2) OR (Pandemics)) AND (("homes for the aged") OR ("nursing homes") OR (residents)) AND (("patient safety") OR (“Visitors to Patients”) OR (“restrictive measures”) OR (“ban for visitors”) OR (“visiting bans”) OR (“social isolation”) OR (lockdown)) AND (("quality of life") OR (wellbeing) OR ("mental health") OR (“acute stress”) OR (depression) OR (“physical health”) OR (“psychological distress”))
